# Impact of wars and natural disasters on emerging and re-emerging infectious diseases

**DOI:** 10.3389/fpubh.2023.1215929

**Published:** 2023-09-01

**Authors:** Seher Topluoglu, Aysegul Taylan-Ozkan, Emine Alp

**Affiliations:** ^1^Provincial Health Directorate of Ankara, Republic of Türkiye Ministry of Health, Ankara, Türkiye; ^2^Department of Medical Microbiology, Medical Faculty, TOBB University of Economics and Technology, Ankara, Türkiye; ^3^Department of Clinical Microbiology and Infectious Diseases, Medical Faculty, Ankara Yildirim Beyazit University, Ankara, Türkiye

**Keywords:** emerging infectious diseases, re-emerging infectious diseases, wars, natural disasters, public health

## Abstract

Emerging Infectious Diseases (EIDs) and Re-Emerging Infectious Diseases (REIDs) constitute significant health problems and are becoming of major importance. Up to 75% of EIDs and REIDs have zoonotic origin. Several factors such as the destruction of natural habitats leading humans and animals to live in close proximity, ecological changes due to natural disasters, population migration resulting from war or conflict, interruption or decrease in disease prevention programs, and insufficient vector control applications and sanitation are involved in disease emergence and distribution. War and natural disasters have a great impact on the emergence/re-emergence of diseases in the population. According to a World Bank estimation, two billion people are living in poverty and fragility situations. Wars destroy health systems and infrastructure, curtail existing disease control programs, and cause population movement leading to an increase in exposure to health risks and favor the emergence of infectious diseases. A total of 432 catastrophic cases associated with natural disasters were recorded globally in 2021. Natural disasters increase the risk of EID and REID outbreaks by damaging infrastructure and leading to displacement of populations. A Generic National Action Plan covering risk assessment, mechanism for action, determination of roles and responsibilities of each sector, the establishment of a coordination mechanism, etc. should be developed.

## 1. Introduction

Emerging Infectious Diseases (EIDs) are illnesses that are newly defined or have existed but are increasing in incidence or geographic range which poses a threat to the population, either in a particular area or globally (1–3). Conversely, Re-Emerging Infectious Diseases (REIDs) are illnesses that existed in the past but reappear after they have been on a significant decline, and rapidly spread either in terms of incidence or to new geographical areas (2–5).

Many factors such as the destruction of natural habitats, which leads humans and animals to live in close proximity, changing climate and ecosystems, replacing reservoir hosts or intermediate insect vectors, and microbial genetic mutations are involved in the emergence of new diseases (6). Ecological changes due to natural disasters such as flood/drought and earthquakes, population migration resulting from war or conflict, interruption or decrease in disease prevention programs, and insufficient vector control applications and sanitation have great importance in disease emergence (1). Environmental and social determinants resulting from natural disasters, wars, etc. are also of great importance in disease re-emergences as well as viral and microbial factors (4). Major contributing factors to the prevalence of EIDs and REIDs are summarized in Table 1.

**Table 1:** Selected major and contributing factors to the prevalence of emerging and re-emerging infectious diseases [Adopted from Church (7)].

Major factors	Contributing factors
Global changes in population demographics and distribution	Population growth and density, migration to urban areas, increased widespread travel, immigration, and housing practices
Change in human behaviors	Food distribution and transportation services, liberation of sexual practices, increased need for childcare outside the home, alcohol and drug abuse, change in immunization practices
Changes in environment and land usage	Global climate changes such as increase in average temperature, deforestation, change in land usage, and natural disasters (Floods, intense droughts, catastrophic storms)
Chronic manifestations of infectious diseases	Modern medical technology in developed countries prolongs the life of people with life-threatening chronic diseases
Advanced pathogen detection	Advanced molecular methods have been developed for the detection of fastidious, uncultivable organisms
Microbial evolution	Adaptation of microorganisms to the environment in order to survive
Collapse of public health systems and bioterrorism	Decreased funding of the public health systems, insufficient public health infrastructure, mobility of population, increased international travel, immigration and refugees, wars, conflicts, bioterrorism

Re-emergence of diseases that were considered to have ended at the beginning of the 20^th^ century has begun in recent years (8). Wars and natural disasters have a great impact on the emergence/re-emergence of diseases in the population.

## 2. Impact of wars on human health

According to a World Bank estimation, two billion people are living in poverty and fragility situations (9). Wars destroy the health systems and infrastructure, curtail the existing disease control programs and cause population movement. Large population displacement into overcrowded camps and temporary shelters leads to an increase in exposure to health risks such as disease vectors favoring the emergence/re-emergence of infectious diseases (10, 11). Outbreaks of typhoid fever, cholera, dysentery, malaria, small-pox, typhus fever and influenza caused a significant number of deaths among soldiers and civilians in war zones during or after World War I and World War II (12, 13).

After a dramatic increase in morbidity/mortality rates in war-involved countries during and after World War I, tuberculosis was recognized as a war disease and became one of the main concerns associated with wars and conflicts (12, 14, 15). The disease re-emerged in Europe as a result of World War I where the incidence was declining (14). As most of current conflicts are prevalent in tuberculosis-endemic countries, tuberculosis continues to be an important threat not only to the people living in conflict areas, but also to the population living in unaffected areas and neighboring countries since huge numbers of people move to safer places internally or internationally (14–17).

One of the examples is the Syrian crisis which led millions of people to migrate to neighboring countries such as Türkiye. The proportion of foreign-country-born tuberculosis cases among total reported cases in Türkiye increased progressively from 1.1% in 2010 to 6.0% in 2014, 7.3% in 2017, and 10.84% in 2018. 587 Syrian cases constituted 53.0% of foreign-country-born cases and 4.87% of the total cases diagnosed in Türkiye in 2017 (18, 19).

The possibility of death in tuberculosis patients who received irregular or no treatment during the conflict was up to three times higher than the ones who received a full course of treatment during peacetime in Guinea-Bissau, West Africa (20, 21).

In a study assessing the impact of war on Ebola transmission and control in the Democratic Republic of the Congo (DRC), it was found that an increase in the number of cases had been observed over and over due to conflict conditions. Violence against healthcare workers and Ebola treatment centers inhibited the rapidity of case isolation, treatment, following up the contacts of cases, and vaccination programs due to continuous conflict events (22). War in the DRC contributed also to an increase in the transmission of sleeping sickness (23), river blindness (22, 24), and pneumonic plague (25–27) in addition to the spread of Ebola.

Destruction of healthcare infrastructure during wars resulting in weakened prevention and treatment programs can cause new strains of infectious diseases to emerge (28). Eradication of Guinea worm, river blindness, and polio programs have been disrupted resulting in challenges to delivering vaccines due to insecurity and conflict in countries experiencing war (28, 29).

As of 20 March 2023, around 8.1 million Ukrainians have moved to other European countries since 24 February 2022 after the attack by Russia on Ukraine, 5 million of whom were recruited into protection programs (30). Owing to the living conditions in the temporary shelters and the conditions they had faced during their movement, these displaced people are likely to acquire certain infectious diseases including EIDs or REIDs, and cases of EIDs or REIDs among this population are not unexpected (31).

There have been numerous reported outbreaks of conflict/war-associated EIDs/REIDs from a wide range of countries. Selected outbreak reports are presented in Table 2.

**Table 2:** Selected EIDs/REIDs outbreaks associated with wars.

Year	Continent/Region	EID/REID	Summary
Africa			
1998	DRC	African trypanosomiasis	Because of the weakness of many general health services which seriously hamper the integration of control activities, the annual number of reported sleeping sickness cases rose from 5,825 in 1991 to 26,318 in 1998 (23).
2005	Angola	Marburg	Nine Marburg hemorrhagic fever cases were diagnosed on 21 March, 2005 by the Centers for Disease Control and Prevention (CDC) in the samples sent from Angola. Being the first natural outbreak of the disease to take place in an urban setting, 270 cases with a case fatality rate of 92 percent were reported within the outbreak. Almost three decades of civil unrest have created additional challenges in the effort to contain the outbreak (32).
2006	DRC	Plague	Several plague outbreaks have been reported in DRC. 100 cases of suspected pneumonic plague, including 19 deaths were reported on June 2006 (27).
2020–2021	DRC	Plague	Implementation of control measures had been difficult due to security concerns in the area. More than 400 cases have been reported during outbreaks in 2020 and 2021 which were aggravated by conflict, poverty, increased population movement and displacement, and instability in some areas (25, 26).
2020	Guinea	Yellow Fever	A yellow fever outbreak with 50 suspected cases was reported in Guinea in 2020. The yellow fever vaccination coverage was found to be 16% in the Koundara district which was one of the affected areas while the estimated vaccination coverage against yellow fever for the whole country had been 40% for the years 2016 to 2019 (33).
2022	DRC	Ebola	EVD re-emerged in the Beni health zone, which was affected by insecurity from armed groups, DRC, 21 August 2022. Frequent protests against the security measures put in place by the authorities and against international organizations increased the risk of refusal of outbreak control measures (34).
2022	Malawi	Polio	Malawi reported a wild poliovirus type 1 (WPV1), which was later shown to be genetically linked to a Pakistan sequence detected in 2020 in Sindh province, in February 2022. The detection of WPV1 outside Pakistan, where the disease is endemic demonstrated the continuous risk of international spread of the disease (35).
Asia			
1994	Tajikistan	Malaria	The number of laboratory-confirmed malaria cases increased from 175 in 1990 to 2,400 in 1994 in Tajikistan during the civil strife in 1992–1993 which caused massive internal displacement of people combined with deteriorating living conditions, and paralysis of the health system, especially public health programs. Additional to the major malaria outbreak, falciparum malaria transmission in some areas of the country was re-established for the first time in 35 years (36).
1997–1998	Pakistan	Cutaneous Leishmaniasis	A major cutaneous leishmaniasis outbreak was seen in an Afghan refugee camp in a non-endemic area in north-western Pakistan in 1997–1998. Before the outbreak, there were only a few cases in the camp, which was hosting more than 9,000 residents, mostly from eastern, central, and northern Afghanistan. Additionally, this outbreak caused disease establishment in the neighboring Pakistani villages (37).
2002	Afghanistan	Malaria	There had been a reemergence of malaria in Afghanistan with an estimation of 3 million cases in 2002 of which most of them in Kundoz Province. The annual incidence of *Plasmodium falciparum* and *Plasmodium vivax* malaria fluctuated from 0.0088 to 4.39 and from 3.58 to 13.37 episodes per 1,000 person-years, respectively. The introduction of *P. falciparum* and *P. vivax* malaria by returning refugees was one of the contributing factors (38).
2012	Lebanon	Leishmaniasis	The annual number of leishmaniasis cases reported in Lebanon sharply increased to 1,275 in 2012 due to mass population migration from Syria. All the cases were Syrian refugees from Aleppo, where they had been infected, causing re-emergence of the disease in the country. Lebanon reported ranged between 0 and 6 cases with no local transmission before 2012 (39).
Europe			
2000	Kosovo	Tularemia	A tularemia outbreak with 327 cases in 2000 was seen in Kosovo as a result of environmental disruption, mass population displacements, the collapse of public health functions, such as disease surveillance and outbreak response, and a breakdown of sanitation and hygiene due to more than 10 years of political crisis and warfare (40).

## 3. Impact of natural disasters on human health

Natural disasters increase the risk of infectious disease outbreaks, including EIDs and REIDs, by damaging infrastructure and leading to displacement of populations (41). The population residing in natural disaster-prone areas has risen in most countries due to land shortage, urbanization, poverty, and population growth, and led to an increase in the public health impacts of natural disasters (42, 43).

A total of 432 catastrophic cases associated with natural disasters were recorded globally in 2021 though the average was 357 between 2001 and 2020 (44). The annual number of floods, which is the most common event among natural disasters, has increased from an average of 163 in the 2001–2020 period to 223 in 2021 (44). Additionally, globalization, climate change, and population movement cause natural disasters and their effects to become more complex (41).

Natural disasters are stratified into several categories by experts. Even though one classification divides natural disasters broadly into three general types (hydrometeorological, geophysical, and geo-meteorological disasters), any disaster in one category can have elements of another, for example earthquakes can cause avalanches and tsunamis, which can, in turn, cause flooding (45).

Hydrometeorological disasters such as floods from rains, hurricanes, cyclones, typhoons, and tsunamis are the most common natural disasters followed by geophysical disasters (Earthquakes, volcanic eruptions) while geo-meteorological ones are rarer than the others (45). Sometimes an emergence or re-emergence of an infectious disease agent can be, by itself, a natural disaster. Centre for Research on the Epidemiology of Disasters (CRED) classify these epidemics/pandemics as a biological sub-group of natural disasters (46). Plague outbreaks, especially the 14th century Black Death period and more recently the emergence of SARS-CoV-2 are the best examples. Even though these diseases cannot be attributed to any specific war or natural disaster they did have serious consequences on the control and prevention of other infectious diseases.

There have been numerous reported outbreaks of natural disaster associated EIDs/REIDs from a wide range of countries. Selected outbreak reports are presented in Table 3.

**Table 3:** Selected EIDs/REIDs outbreaks associated with natural disasters.

Year	Continent/Region	Natural Disaster	EID/REID	Summary
Africa				
1987–1988	South Africa	Flood	Poliomyelitis	An outbreak of type 1 poliomyelitis with 23 cases reported triggered by the massive floods, experienced in the area 2 months earlier in the eastern part of South Africa between December 1987 and November 1988. The cases were most probably due to temporary breakdown in vaccination services and considerable surface pollution, including ‘wild’ poliovirus as a result of flooding (47).
2000	Mozambique	Flood	Malaria	After the heavy rain in Mozambique in 2000 the incidence of malaria increased by four to five times compared to the same period in other years (48).
America				
1991	Costa Rica	22 April 1991 Earthquake	Malaria	After the earthquake on 22 April 1991, measuring 7.4 on the Richter scale, statistically significant increases in the incidence of malaria were reported during the months immediately after the earthquake in Limon Province. The number of registered malaria cases for 1 June through 31 May period dramatically increased from 681 in the 1990–1991 period to 3,597 on 1 June 1991 through 31 May 1992 in the same region (49).
1991	USA	Drought	Leptospirosis	Five leptospirosis cases associated with swimming in a rural swimming pond were reported from a small town in rural Illinois between July 7 and 18 in 1991. The outbreak was attributed to the drought conditions creating an environment in the pond facilitating transmission of the organism from area animals to humans (50).
1993	USA	Flood	Leptospirosis	Following the floods in 1993, leptospirosis cases exposed to extensive flood water were reported in Iowa (51).
1994	USA	1994 Northridge, California Earthquake	Coccidioidomycosis	A coccidioidomycosis outbreak between 24 January and 15 March was reported in Ventura County following the January 1994 earthquake, centered in Northridge, California. It is suggested that the outbreak was caused by arthrospores spread in dust clouds generated by the earthquake (52).
1996	Brazil	Flood	Leptospirosis	An increase in the number of leptospirosis cases was reported during the subsequent weeks, after the heavy rainfall caused persistent flooding in several areas of Rio de Janeiro accounting for the largest epidemics in the city’s history. The incidence of leptospirosis was around 1 case per 100,000 inhabitants in the city of Rio de Janeiro yearly before the outbreak. The disease incidence fluctuated to 42.05 per 100,000 inside the flood-risk area (53).
1998	Nicaragua and Guatemala	Hurricane Mitch	Cholera, Leptospirosis and Malaria	Hurricane Mitch in 1998 affected several countries in Central America which damaged health services, wate,r and sanitation networks, and caused population movements between neighboring countries. Thirty-eight cholera outbreaks with 33 deaths in Guatemala, and a leptospirosis outbreak in Nicaragua with 7 seven deaths were seen. Also, the number of reported malaria cases was much higher than the weekly average reported during the pre-Mitch period in Nicaragua and Guatemala (during the second and third weeks) (54).
2001–2003	USA	Drought	West Nile virus	Sporadic and focal transmission of West Nile Virus (WNV) in humans and sentinel chickens has been reported from Florida between 2001–2003. Transmission of WNV was associated with drought 2–6 months prior and land surface wetting 0.5–1.5 months prior probably due to drought that brought avian hosts and vector mosquitoes into close contact facilitating the epizootic cycling and amplification of the arboviruses within these populations (55).
2003–2011	USA	Drought	West Nile virus	Analysis of the field surveys of local mosquito communities and the prevalence of WNV within *Culex* spp. populations for transmission seasons of 2012 and 2011, and the WNV infection rates and climate data from nine transmission seasons (2003–2011) in New Jersey showed drought conditions (i.e., increased temperatures and decreased precipitation totals), were associated with increases in the prevalence of WNV and confirmed that climatic conditions have a strong impact on the prevalence of vector-borne diseases (56).
2004	Dominican Republic	Flood	Malaria	After the Hurricane struck the Dominican Republic, the east coast of the country received heavy rains and flooding resulting in increased mosquito breeding sites. The number of malaria cases increased sharply, and 2,012 cases had been reported within one month in 2004, which was approximately 1,500–2,500 cases annually in the country (57, 58).
2004	USA	Flood	Leptospirosis	After a flood in a university campus, two leptospirosis cases, of which one was a professor cleaning his flooded laboratory in sandals, were diagnosed (59).
2005	Guyana	Flood	Leptospirosis	The widespread flooding following the unusually high rainfall in January along the Atlantic coast led to conditions favorable for epidemic leptospirosis. An outbreak of leptospirosis associated with flooding was confirmed *via* laboratory testing (60, 61).
2010	Haiti	Earthquake on January 12, 2010	Malaria	After the great earthquake, 11 laboratory-confirmed cases of whom seven of them were emergency responder U.S. residents, of *P. falciparum* malaria acquired in Haiti reported (62). By enhanced surveillance conducted between 4 March and 9 April, 2010, 317 more malaria cases were diagnosed most probably due to displaced persons living outdoors or in temporary shelters, putting them at increased risk for acquiring malaria (63).
2011	USA	Tornado	Mucormycosis	After the tornado on 22 May, 2011, a large cluster of cases of mucormycosis, with 13 *A. trapeziformis* infections in persons injured during a tornado reported, suggesting environmental fungi be considered as potential agents of soft-tissue infections in injured patients after disasters (64).
2016	Ecuador	Earthquake on April 16, 2016	ZIKV	A significant increase in the number of Zika Virus (ZIKV) cases was seen in the affected areas after the 7.8 magnitude earthquake in Ecuador on 16 April, 2016 (65, 66). The total number of ZIKV cases in the country rapidly escalated from 92 to 1,106 in just 3 months. Eighty percent of the cases were reported from the region most affected by the earthquake (67).
Asia				
1997–1998	Indonesia	Drought	Malaria	A dramatic increase in malaria and an associated 550 deaths were reported beginning in late August 1997 due to the prolonged and severe drought created by the prevailing 1997–98 El Niño Southern Oscillation (ENSO) in Irian Jaya, Indonesia. Drought conditions resulting in numerous, transient pools of standing water permitting a rapid increase in vector populations and movement and exposure of the population to high-risk malaria endemic lowlands were the main drivers of the outbreaks (68).
1999	Japan	Heavy rainfall	Leptospirosis	After the heavy rainfall in 1999, an outbreak of leptospirosis in the Yaeyama Islands was reported. Fourteen people who were exposed to contaminated soil or water were diagnosed with leptospirosis and required hospitalization (69).
2000	India	Flood	Leptospirosis	Two weeks after heavy rains led to floods in Mumbai in July 2000, an outbreak of leptospirosis was reported in adults admitted to public hospitals. Additionally, leptospirosis was diagnosed in 18 children who were admitted to one hospital between 24 July and 14 September 2000. All of them had contact with flood water (70).
2003	Iran	December 2003 Bam city Earthquake	Cutaneous leishmaniasis	After an earthquake created 10 million tons of rubble, creating suitable conditions for the propagation of sand fly vectors in Bam city of Kerman province in Iran, 2003, a new Anthroponotic Cutaneous Leishmaniasis (ACL) focus in the villages of Dehbakry county, of which there had not previously been any record of CL, was established and an outbreak reported in November 2004 (71).
2003	Iran	July 2003 Fars Earthquake	Cutaneous leishmaniasis	The annual incidence of CL in Fars, Zarindasht, increased from 58.6 cases/ 100,000 in 2002 to 864/100,000 in 2004 after the two earthquakes on 10 July 2003. The cases were predominantly Zoonotic CL (65).
2003	Iran	December 2003 Bam city Earthquake	Cutaneous leishmaniasis	Creating various risk factors; the earthquake caused a sharp increase in the incidence of anthroponotic cutaneous leishmaniasis (ACL) cases in Bam. The mean annual incidence of ACL increased from 1.9 per 1,000 for the period of 1999–2003 to 7.6 per 1,000 for the period of 2004–2008 (66).
2004	Indonesia	Tsunami	Melioidosis	Ten patients with pneumonia of whom four were with culture-confirmed melioidosis, were diagnosed among tsunami survivors as a result of immersion in contaminated saltwater during the tsunami (72).
2007	China	Flood	Malaria	Several floods occurred following the persistent and heavy rain in June and July in the Huaihe River Basin in 2007. The monthly mean monthly malaria incidence fluctuated from 13.76/10 to 95.78/10 in the area. Increased risk of malaria was significantly associated with flooding (73).
2009	Taiwan	Typhoon	Leptospirosis, melioidosis	After Typhoon Morakot in 2009, unusual epidemics of leptospirosis and melioidosis cases were reported. Incidences of leptospirosis and melioidosis cases after the typhoon were significantly higher than those before the typhoon (74).
2011	Sri Lanka	Flood	Leptospirosis	In 2011, a large outbreak of leptospirosis with 32 cases was observed in Anuradhapura district, which was not previously classified as a leptospirosis endemic area, after two weeks of massive flooding (75).
2015	Nepal	Earthquake	Scrub typhus	Three months after the devastating earthquake in April 2015 in Nepal; the first and most significant fatal scrub typhus outbreak was reported in the country with 141 cases in 2015, and lasted for three years (76–78).
Europe				
1997 and 2002	Czech Republic	Flood	Leptospirosis	Leptospirosis has been reported rather sporadically, with an incidence rate of about 0.3% per 100,000 population in the Czech Republic. After vast floods in 1997 and 2002, the incidence of leptospirosis increased to 0.9 per 100,000 population (79).
1999	Türkiye	17 August 1999 Earthquake	Tularemia	After the 1999 earthquake, an oropharyngeal tularemia outbreak was reported from the Golcuk-Kocaeli region beginning with 5 cases followed by 129 more cases. This was the first outbreak in the region and most of the cases were from the houses located around natural springs in the new settlement constructed after the earthquake. The outbreak was probably due to pollution of natural springs infected by wild rodents or other infected animals (80).
2004	Finland	Tsunami	Melioidosis	Melioidosis cases were reported in survivors of tsunami that occurred in coastal areas of the Indian Ocean rim in December 2004. *Burkholderia pseudomallei* was isolated from three Finnish patients in January 2005 whom were visiting Thailand when the tsunami struck in December 2004 (81).
2010	Austria	Flood	Leptospirosis	Four leptospirosis cases were reported in athletes after a triathlon held in Langau, a village in Austria, due to intense rain on the eve of the triathlon that had caused flooding and “unusual turbidity” of the lake (82).

## 4. Drivers of emerging and re-emerging infectious disease outbreaks in wars and natural disasters

There are several causal factors in emerging and re-emerging infectious disease outbreaks in wars and natural disasters. Population movement and migration, environmental health disruption and ecological changes, displacement of domestic and wild animals, the collapse of health systems and disruption of disease control programs, inadequate surveillance and early warning and response systems, impaired laboratory services and diagnosis, and breakdown in infection control and treatment in effectiveness and development of drug resistance are the most accepted drivers of emerging and re-emerging infectious disease outbreaks in wars and natural disasters.

The impact of wars and natural disasters on emerging and re-emerging infectious disease outbreaks can be direct or indirect.

## 5. Direct impact with increasing conditions for vector/reservoir proliferation or environmental conditions

Waterborne, rodent-borne, and vector-borne diseases are the main groups of diseases associated with natural disasters. Emergence and increase in the incidence of waterborne diseases due to *Campylobacter*, *Cryptosporidium*, *Escherichia coli*, *Giardia*, Hepatitis A virus, Norovirus, *Shigella,* and *Salmonella* are generally reported from the affected areas (83).

It is widely recognized that natural disasters, especially hydrometeorological ones like floods, hurricanes, and cyclones, have the potential to lead to an increase in vector-borne diseases due to environmental factors (enlargement of vector habitats in number and area) enhancing vector population densities (84, 85). Existing mosquito breeding sites may be washed away at the beginning of flooding, but later on, new breeding sites for mosquitoes can occur due to overflows of rivers or rainfall and therefore enhance the risk of re-emergence of mosquito-borne diseases such as malaria, Chikungunya, and dengue (57, 85–87). There have been also emergence/re-emergence and outbreak reports of Chikungunya virus (CHIKV), Tahyna virus (TAHV), West Nile virus (WNV), and Japanese encephalitis diseases linked to flooding events (83, 88). Flooding can also cause a proliferation of disease vectors and microorganisms by changing the balance in ecosystems and the environment (43, 86). Mosquito-borne diseases, related to ecological changes which are suitable for mosquito breeding, also increase after earthquakes (49). Following the devastating earthquake in Ecuador in 2016, an important increase in the incidence of Zika Virus (ZIKV) was observed in the affected regions (89, 90). After the earthquakes in Iran in 2003, Cutaneous Leishmaniasis (CL) cases fluctuated, new foci emerged and the epidemiology of the disease changed (65, 66, 71, 91).

Mosquito-borne diseases were also reported during drought conditions. Drought causes reduce in the size of water bodies making them stagnant which leads to additional breeding places for mosquitoes (92). Also, the collection of rainwater due to inadequate water supply can increase the stagnant water sources (92). Drought-induced amplifications of the West Nile virus have been reported in the USA (55, 56). Malaria cases and deaths increased excessively due to the drought following an El Niño Southern Oscillation that occurred in Indonesia (68). Drought conditions caused numerous stagnant water puddles in rivers acting as mosquito breeding places (68, 93).

The risk of diseases transmitted by rodents such as leptospirosis and Hantavirus Pulmonary Syndrome increases as the probability of contact with bacteria and their animal hosts rise during heavy rainfall and flooding (94, 95). Flooding catalyzes the transmission of leptospirosis as a result of an increase in the number of infected rodents sheading leptopiras (57, 95). Insufficient garbage collection and management causing the spill of rubbish into streets after natural disasters can lead to an increase in rodent population (96, 97).

Rarely, unusual disease outbreaks such as coccidioidomycosis and scrub typhus have been observed following earthquakes. For example, an outbreak of acute pulmonary coccidioidomycosis related to exposure to high-level airborne dust following landslides after the 1994 earthquake occurred in southern California (52, 98). This outbreak illustrates the relationship between specific environmental conditions and the emergence of infectious diseases (52). After the massive earthquake in Nepal in 2015, scrub typhus emerged and outbreaks were reported from various parts of the country due to rodent infestation of the environment around temporary shelters which led people and rodents to be in close proximity (76, 77, 99).

Also, the destruction of infrastructure such as electricity, water supply, sewage disposal, and gas supply increases the risk of food poisoning and water-borne diseases after natural disasters (100, 101).

Outbreaks of leptospirosis and malaria were reported in Guatemala and Nicaragua after Hurricane Mitch in 1998. The number of diagnosed malaria cases was considerably higher than the number of cases diagnosed before the hurricane (54).

Melioidosis cases were reported in survivors of the tsunami which occurred in coastal areas of the Indian Ocean in December 2004 (72, 81). The risk of waterborne and vector-borne diseases increases due to physical changes in the environment in tropical cyclones, followed by floods and sea surges (102).

Findings from previous El Nino episodes show that rains due to El Nino that result in increased rodent density heightens the risk of human contraction of Hantavirus pulmonary syndrome (103). A previously undescribed disease, subsequently identified as a previously unknown hantavirus (*Bunyaviridae*), emerged in the Southwest USA in 1993 after El Nino Southern Oscillation events the year before, which had allowed excess precipitation, warmer seasons, and plenty of food for rodents (21, 103). Also, a statistically significant association was demonstrated between malaria epidemics and El Niño most probably due to widespread flooding during El Niño (104).

## 6. Indirect impact through changes in human behavior

### 6.1. Population movement and migration

Population displacement is generally observed after natural disasters and wars (105, 106). Population displacement can lead to contact with infectious agents both for the migrating population and for the population at their point of arrival. The migrating population can introduce new infectious agents to the population living in their destination. Conversely, the migrating population can get in touch with new local pathogens. Additionally, the importation of the diseases from endemic regions to non-endemic regions by the migrants (107, 108).

According to UN Refugee Agency, 89.3 million people—of which 27.1 million were refugees—had to leave their homes in 2021 because of conflicts/war, assault, human rights violations, etc. Around 69 percent of people displaced across borders originated from Afghanistan, Myanmar, South Sudan, the Syrian Arab Republic, and Venezuela. The Syrian refugee population constituted 27 percent of the global refugee population with around 7 million and spread to 129 countries. Most of them are hosted in Türkiye (3.7 million), Lebanon (840,900), and Jordan (673,000) (109).

The displaced population is generally housed in temporary settlements or camps with overcrowded shelters, which increases infectious disease transmission and the number of endemic and epidemic-prone disease cases (11, 85). There have been numerous reports of EIDs/REIDs related to population movement associated with the destruction of health services and disease control programs.

Being the largest refugee crisis since World War II, the Syrian crisis is one of the examples of how conflict and refugees change epidemiological patterns of diseases not only in the source country but also in neighboring countries (85, 110). Cutaneous leishmaniasis (CL) has always been reported in Syria, particularly in the western part of the country. CL cases had increased from 17.709 in 2007 to 78.231 (suspected cases 111.144) in 2021 due to decreased public health services, reduced diagnosis and treatment access, population displacements, demolition of infrastructure, and worse living conditions as a result of war. Additionally, the epidemiology of the disease in Syria also changed during wartime; mass population movements from highly endemic areas raised the number of cases in some governorates, which used to report few cases before (111–113). Also, the CL situation in countries that hosted huge numbers of Syrian refugees altered. The disease re-emerged in Lebanon and the number of cases rose to 1.383 in 2014 (39, 114). Even though CL is endemic in Türkiye, the number of CL cases increased from 1.133 in 2008 to 5.362 in 2013 (115).

The risk of outbreaks of norovirus, *Salmonella enterica*, and chickenpox resulting from post-disaster living conditions increases after earthquakes (41, 100). Diarrhea and respiratory tract infections are the most common diseases reported after earthquakes (116, 117).

Movement of workers from the CL endemic regions to Bam city in order to work on construction projects, provision and creation of suitable conditions for propagation of sand fly vectors and many new resting and breeding sites, changes in the behavior of people following earthquake increasing their exposure to the bites of the vectors, and displacement of the wild mammals acting as the ‘reservoir’ hosts of the parasite (65, 66, 71, 91) were likely to be the main drivers.

In drought conditions both human and animal behavior changes, such as movement to places where water supplies can be found. This increases the likelihood of contact with one another (92, 93). Current theories suggest even the bubonic plague outbreaks in Europe in the 14^th^ century were caused by the drought in Asia which led the introduction of infected rodents to Europe (118, 119).

### 6.2. Displacement of domestic and wild animals

During natural disasters, war, or conflicts animals can be lost, abandoned, or left unsupervised leading to a high number of stray or owned free-roaming dogs (120). Free-roaming dogs and cats are source of a variety of bacterial, viral, and parasitic infections which pose risks to humans (121). Free-roaming dogs change or increase the risk for emergence and transmission of zoonotic diseases in dogs and people such as rabies, echinococcosis, toxocariasis and even leptospirosis, Chagas disease, and leishmaniasis (122, 123).

Colonization of rodents can be increased by dog foraging activities in accumulated waste in camps (122, 124). Loss of rodent control resulted in the Lassa outbreak in refugee camps in Sierra Leone (106).

The risk of rabies can be increased due to a higher roaming dog population, increased aggression in dogs and dog bites, reduced/low vaccine rates in dogs, and post-exposure prophylaxis in humans (122) in natural disasters and wars. There have been some studies suggesting an increase in the incidence of dog bites following natural disasters (125–128). News stated that cases of exposure to dog bites have increased in the areas of northwestern parts of Syria and rabies cases were reported in 2022 (129).

An increase in contact with wildlife due to population movement is another risk factor for EIDs/REIDs in natural disasters and especially in conflicts (130). Disruption of livelihoods that is likely to increase the consumption of bush meat (131, 132) and increased exposure to animal hosts such as bats, increase the likelihood of zoonotic diseases (106, 130). Evidence suggested that Ebola may have been spread by these population movements in conflict areas (130).

European Centre for Disease Prevention and Control (ECDC) warns the countries accepting Ukrainians about rabies risk as it is still endemic in sylvatic animals and dogs and cats in Ukraine due to the reports describing displaced Ukrainians as fleeing with their pets (31).

## 7. Indirect impacts through the collapse of health systems and sanitation services

### 7.1. Collapse of health systems and disruption of disease control programs

The deterioration of the public health systems leads to an interruption in health measures such as immunization, surveillance, prevention and control programs, sanitation, and hygiene services and results in an increase in communicable diseases (105, 133). Disruption of public and animal health care systems has a major impact on the increase in vulnerability of displaced populations to different kinds of infections (85). Beyond the destruction of pre-existing local health facilities such as buildings, medical stores, and laboratories, access to functioning facilities can be blocked due to risks involved in traveling through the conflict zones (11, 134). Moreover, local medical staff are often also affected by the conflict or natural disaster, making them incapacitated (133).

The incidence of vector-borne diseases such as malaria can increase due to the disruption of control activities (135). Re-emerge of malaria in former Soviet Union republics is an excellent example of how the disruption of disease programs due to conflict can alter the disease epidemiology.

Malaria had been eradicated in all the republics of the former Soviet Union with a campaign launched at the end of the 1950s (136). Re-emergence of malaria started in the former Soviet Union republics due to internally displaced people and mass population movement from malaria-endemic neighboring countries, mainly Afghanistan, experiencing armed conflicts and war in the 1990s (137). Beyond this; the deterioration of preventive measures including malaria services due to economic collapse as a result of the disintegration of the former Soviet Union contributed to the situation (137). Malaria re-emerged in Armenia, Azerbaijan, Georgia, Kazakhstan, Kyrgyzstan, the Russian Federation, Tajikistan, Turkmenistan, and Uzbekistan and several outbreaks have been reported afterwards (137). During the civil war in 1992–1993, with the influx of massive population displacement united with deterioration in living conditions and breakdown of the health system, particularly public health programs, and refugees from Afghanistan, a major malaria outbreak reached 29.794 cases in 1997 in Tajikistan, where malaria had been eradicated in 1960. Moreover, *P. falciparum* malaria was re-established after 35 years in Tajikistan due to mass population movement to and from Afghanistan (36, 137).

Curtailment of vaccination programs is especially crucial. A decline in or suspensions of vaccination programs have serious effects not only on the local disease programs but also globally implemented ones. Conflicts are now causing re-emergences of diseases that were in the eradication phase such as polio (138). The World Health Assembly put in force a resolution for polio eradication globally in 1988 (139). Due to conflict the three doses of polio vaccine coverage dropped to 35% and led to a large polio outbreak in Somalia in 2005 (10, 140).

The last case of wild poliovirus in Syria had been reported in 1999 (141). But in 2013 the disease re-emerged and 36 children of whom most had not been vaccinated against polio were paralyzed (141). In addition, another poliovirus outbreak in 2017 left 74 children paralyzed in Syria (142). The emergence of poliovirus in Syria manifests the public health results of the war (143).

The last case of autochthonous wild polio was reported in 2020 in Africa, and in Malawi the last clinically confirmed wild polio case was reported in 1992 (35). Wild poliovirus type 1 (WPV1) cases were diagnosed in Malawi and in Mozambique on 17 February 2022 and 15 May 2022, respectively, which the WHO considered at high risk of spreading, particularly to the countries of Southeast Africa, owing to their low immunity and surveillance, huge population movements, and decreased immunization rate (35, 144).

Yellow fever outbreaks were reported in West Africa as a result of the curtailment of immunization programs and spontaneous or forced migrations of thousands of people due to conflict. The circulation of the yellow fever virus in Africa has increased significantly between 2000 and 2004 (10, 145). Yellow fever re-emerged in 2020 with two outbreaks in West African countries (Guinea and Senegal) and enlarged to 12 African countries of which most have been facing political instability and insecurity (146). The outbreak continued in nine countries of the WHO African Region (Central African Republic (CAR), Cameroon, Chad, Côte d’Ivoire, DRC, Ghana, Niger, Nigeria, and the Republic of Congo) in 2021 with aggregation of decreased routine immunization and increased population movement (147). A resurgence of yellow fever began additionally in four countries (Kenya, Niger, Sierra Leone, and Uganda) in 2022 (148).

Security problems are another concern in wars, and can hinder the implementation of control programs and also can cause delays in response in cases of disease outbreaks (10).

There have been various reports of outbreaks of plague in DRC. A total of 8,379 cases have been reported between 2004 and 2014 in the country (149). The disease re-emerged in 2020 (25, 26) and the outbreak continued in 2021 (26) and 2022 (150). Worryingly, cases are also reported from areas that have not seen cases for several decades (26). The collapse of control programs impeded the access of the population to health facilities, the insecurity of the area, and population displacement due to a prolonged and escalating conflict situation were the main drivers of the outbreaks (26, 149–151).

### 7.2. Insufficient surveillance and impaired early warning and response systems

An effective disease surveillance system is essential in order for timely detection of infectious disease outbreaks both in human and animal populations (85, 152). Surveillance is needed to assess the situation of diseases, follow up the alterations in epidemiology, characterize health risks, trace the populations’ health, guide immediate and long-term measures, prioritize of financial resources for health, and complement more targeted epidemiological and laboratory investigations (153). However, surveillance systems are often weak and adversely affected in conflict situations and disasters or are not designed to serve emergency preparedness and response activities, give early warning of diseases, or gather information necessary to assess needs (10, 152, 153).

Existing surveillance systems may not be functioning or reachable due to the conditions (154). For example, in the earthquake in Haiti in 2010, the identification of the emergence of cholera took several days. The surveillance system and infectious diseases control programs took 2 weeks to be set up following the earthquake (154, 155).

### 7.3. Impaired laboratory services and diagnosis

Laboratory services and facilities face many constraints in disasters and conflict areas including getting the affected region, deficiency in reagents and equipment, limited electrical power and utensil supplies, inadequate personnel, and ineffective supervision. In some situations, laboratories themselves may be in a state of emergency (156, 157). Security risks, damaged infrastructure, and discontinuity in supply chains due to blockades are additional burdens in laboratory systems in conflict-affected areas (158).

Disease surveillance is one of the important components of disaster assessment and monitoring the effectiveness of interventions (156). Diseases that have typical clinical presentations, such as measles, do not require laboratory investigations for diagnosis however most infectious diseases need laboratory facilities to make or confirm the diagnosis (156). Timely and accurate diagnosis is essential in conflict situations and disasters in order to realize disease clusters or even outbreaks, as well as generate data to manage public health interventions (158).

The role of laboratory services in disasters is the prevention or control of infectious diseases, by identification of the causative agent(s) of outbreaks which is especially important in effective disease/outbreak control and the management of conditions that occur secondary to the prime cause of the outbreak/disaster (21, 156). The decrease in etiologic diagnosis can cause an increase in broad-spectrum antibiotics usage which can contribute to the emergence of antimicrobial resistance (21).

Displaced persons or refugees are generally at great distance from access to laboratory facilities (156) which makes them more vulnerable to disease outbreaks. Testing for different pathogens, particularly water-borne ones is especially important in these groups with poor hygiene and overcrowding in order to manage disease outbreaks (158).

The debilitating earthquake in 2005 in Pakistan posed a unique problem with the supplies for the maintenance of laboratory services. The health management in affected areas was almost paralyzed and the existing healthcare system was completely demolished leading to total disruption of the primary and secondary healthcare system. Additionally, health staff became vulnerable due to landslides and weather conditions. The establishment of diagnostic laboratory services was a unique and special challenge. Unavailable appropriate shelters specified for laboratory equipment caused to work in uncontrolled conditions. The efficiency of sensitive equipment was affected due to drastic changes in temperature and electricity supply as the generators were limited. There was a great problem with effective logistic and technical support, and the supply of reagents and diagnostic kits (159). The weak health system including insufficient laboratory diagnostic capacity and a lack of experience in patient treatment and community awareness resulted in the further spread of Ebola in Sierra Leone in 2014 (160). Patients died due to the late laboratory confirmation, leading them to not being transported to treatment centers. Additional problems regarding inappropriate specimen delivery systems, a lack of consistent electricity which resulted in broken laboratory equipment and water supply, and the disruption of instruments owing to high temperature and humidity had been also experienced during the outbreak (160).

### 7.4. Breakdown in infection control

Breakdown in infection control in natural disasters and wars is generally observed. The principles of infection prevention and control (IPC) remain especially important given the disrupted health services, insufficient trained staff and personal protective equipment, and the unhygienic circumstances after disasters, which can enable the amplification of infectious diseases. Infection control is especially important in areas where there is a transmission risk of infectious diseases such as Ebola, Middle East Respiratory Syndrome (MERS) (10, 138).

Staff of healthcare centers are at high risk of contracting and transmitting the diseases to their family and the community (161, 162). Prior to infection control procedures, the relative risk of healthcare workers contracting Ebola during the outbreak in West Africa was approximately 100 times higher than in the general population (162, 163). Nosocomial transmission played a major role in two Ebola outbreaks in conflict-affected countries, the DRC in 1995 and Uganda in 2000 (10), most probably from a breakdown in infection control. Another nosocomial outbreak of Lassa fever occurred in Sierra Leone in 2004 due to a breakdown of infection control measures due to a weakened health system during war years (10, 164).

A Marburg hemorrhagic fever outbreak was recorded in long-term war affected Angola in 2004–2005. The outbreak was amplified by multiple usage of injectors and multi-dose ampoules in health facilities (10, 32, 165).

### 7.5. Treatment ineffectiveness and development of drug resistance

The collapse of health facilities, impeded access of the population to surviving health facilities, and insufficient quantities of treatment drugs can cause the cessation of continuing treatment regimens and usage of un-prescribed drugs or inappropriate drug regimens and outdated drugs resulting in pathogens’ increase in infectious diseases transmission and resistance to drugs in conflict situations and natural disasters (43, 85, 166).

The emergence of Antimicrobial Resistance (AMR) in microorganisms is a major health problem. It is linked to insufficient health facilities, inappropriate sanitation measures, weak health care, overuse of antimicrobials, and lack of or outdated treatment guidelines (167, 168). Evidence also indicates that increased international travel and migration including forced migration, can contribute to the spread of drug resistance. Displaced populations and migrants may have a higher burden of AMR due to specific factors such as poor living conditions, limited access to good-quality health care or interruption of treatment during their journey, and poor nutrition (167–169).

Studies show that refugees have an elevated prevalence of AMR carriage and infection and significantly higher rates of methicillin-resistant *Staphylococcus aureus* (MRSA) and multidrug-resistant *Enterobacteriaceae* (MDRE) than the host country population (168, 170). A study which compared the AMR levels of refugees and Germans found that the refugee group carried five or more antibiotic resistance genes whereas most Germans carried three or fewer (171, 172). In a study conducted in Türkiye, it is found that MRSA prevalence and extended-spectrum β-lactams (ESBL) positivity rates of Syrian refugees were higher than that of Turkish citizens (172).

The rapid worldwide emergence and spread of *Acinetobacter baumannii* (*A. baumannii*) is one of the major causes of hospital-acquired infections in recent years (173, 174). *A. baumannii* strains are of an especially high concern in military field hospitals and the ones established after natural disasters (173, 175). Drug-resistant or even multi-drug-resistant (MDR) *A. baumannii* are a great issue of concern in patients with war injuries as war-associated wounds are prone to colonization of multidrug-resistant *A. baumannii* (176). Gaining attention during the 2003 and 2005 period military operations in Iraq, since then there have been several reports of warfare-associated MDR *A. baumannii* infections (174, 176–180).

A study indicated that drug resistance, and MDR-TB in particular, was significantly higher in patients from the refugee population than the local population in Kenya most probably due to a poorly functioning TB treatment program and an incomplete course of medication (166). Drug-resistant TB including MDR-TB has emerged in republics of the former Soviet Union after the fall of the Soviet Union due to civil war in most of the countries, leading to a large number of internally displaced persons, the collapse of public health systems and increased poverty and decline in socioeconomic status (181–184). TB remains a major public health problem in Ukraine having the second highest number of TB cases (31000), with an incidence of 71 cases per 100,000 in the World Health Organization (WHO) European Region. Additionally, the burden of multi-drug resistant tuberculosis (MDR-TB) in Ukraine is extremely high (185). Experts warn about the risk of suspension of TB and DR-TB treatment due to war could have significant results, like amplification of drug resistance, continuous transmission of infection, and death (186).

### 7.6. Environmental health services disruption and ecological changes

The impact of both natural disasters and wars creates several public health problems. The deterioration in environmental health services is one of the major problems (187). Alteration in ecology leads to an increase in vector proliferation and the movements of wild and domestic animals pose risks for disease outbreaks (188). Disasters alter the microbial population in the affected area which can cause new ecological interactions (118).

Vector-borne diseases represent a large group within emerging diseases and major causes of morbidity and mortality. Ecological changes and environmental conditions are very important in vector-borne agent transmission (21). The mosquito population is dramatically affected by environmental conditions such as rainfall and flooding (21).

A tularemia outbreak was reported in 1999–2000 during the civil war in Kosovo due to ecological changes, huge population movements, and deterioration of sanitation (40).

Natural disasters can cause disease outbreaks as a result of the contamination of water sources with feces and chemicals, and the living conditions of populations in overcrowded shelters (189).

## 8. Indirect impact through individual vulnerabilities

Malnutrition, which increases vulnerability to EIDs/REIDs, is a common situation observed both in wars and natural disasters, especially in long-term events such as droughts. Food security created by conflicts, civil strife, and extreme weather conditions—particularly repeated droughts—is the main reason for malnutrition in less developed countries (190, 191). According to World Food Programme around 258 million people were acutely food-insecure in 2022 in 58 countries (191). The Greater Horn of Africa including Djibouti, Ethiopia, Kenya, Somalia, South Sudan, Sudan, and Uganda is the worst food-insecure region for decades. A rising trend in food insecurity is being currently observed in the region due to extreme weather events such as drought and flooding, conflict and instability, high food prices, and the socioeconomic impacts of COVID-19 (192, 193).

Food insecurity can cause impairment in the immune system due to malnutrition making people more susceptible to infectious diseases and generally associated with population displacement, overcrowding, the collapse of health system measures such as vector control and vaccination programs, and poor sanitation which poses the risk of infectious diseases outbreaks (192, 194). Particularly women, children, and people living with chronic diseases like human immunodeficiency virus (HIV) and tuberculosis are vulnerable due to food insecurity (192). There are several well-established studies confirming the bidirectional link between HIV and food insecurity: Food insecurity may increase susceptibility to HIV, and HIV may lessen the HIV patient’s ability to make and/or aquire food (190).

Food insecurity can also contribute indirectly to shape, re-shape, or enhance vulnerabilities to EIDs/REIDs, especially in less developed countries. For example, the risk of contracting HIV/AIDS is much higher in women than men now even though men were more likely to contract the disease when the disease emerged (190, 195). The reason for this inequality is a mixture of several biological, cultural, economic, and social factors, especially in less-developed countries. A study assessing the data of 91 less-developed countries indicated that drought-induced food insecurity was directly and indirectly associated with women’s vulnerability to HIV. Women generally were more likely to get a lower amount of food than men, which causes nutrient deficiencies leading to increased susceptibility to infection. Additionally, food insecurity was also associated with social vulnerabilities such as a decrease in women’s access to health facilities and forced engagement in risky sexual behaviors, etc. (190).

A systematic review analyzing 111 studies even suggested an indirect relationship between drought and HIV treatment in African countries. Changes in livelihood and economic conditions due to drought-induced water and food insecurity and population movement appeared to affect HIV treatment adherence according to the review (196). Another review’s findings identified a relationship between food insecurity and initiation of antiretroviral therapy (ART) and treatment adherence (197).

## 9. Conclusion

The impact of wars and natural disasters on EIDs/REIDs varies depending strongly on the level of underlying fragility or vulnerability in the affected country or society. Less developed countries are significantly more vulnerable to disasters due to having a large number of vulnerable populations, limited response capacity, weak health systems, etc. (198). The inadequate health infrastructure and insufficient human and financial resources before, during, and after wars and natural disasters in less developed countries may increase the vulnerability, and EIDs/REIDs incidences such as malaria and HIV reach epidemic proportions (198). In contrast, EIDs/REIDs outbreaks can be confined within a particular area in developed countries as a result of strong health systems and their coping capacity with disasters.

However, development is not a guarantee of ‘invulnerability,” different levels of vulnerabilities within societies or populations even in developed countries can also be observed (198). The older adults and poor black populations were a significantly high proportion among victims of Hurricane Katrina in New Orleans in 2005 (199, 200). Similarly, the young, the older adults, and women were affected more than others during the earthquake in Italy in 2009 (200).

Conflicts also can generate additional vulnerabilities during natural disasters owing to weakening the respond capacity of countries. Prevailing vulnerabilities in conflict situations are generally aggravated by disasters and conflicts may worsen the impact of disasters. There are some studies suggesting that conflict has been higher in drought situations (201).

In a systematic review analyzing 132 studies, population displacement was the most frequently reported risk factor in infectious disease outbreaks followed by water, sanitation, and hygiene (WASH), housing, and vector/animal after disasters (202). In another review crowded conditions, forced displacement, poor quality shelter, poor water, sanitation and hygiene, lack of healthcare facilities, and lack of adequate surveillance were found to be the key risk factors cascades for communicable disease outbreaks in complex humanitarian emergencies (203). All these factors exacerbate the pre-existing vulnerability and increase the probability of post-disaster infectious disease outbreaks (202).

The extent (geographically, number of people affected, duration, etc.) of the disaster is also important in the context of the coping capacity of the country. For example, the devastating earthquake on 6 February 2023 with a 7.8 magnitude followed by 5,700 aftershocks directly affected 11 provinces living 15 million people in Türkiye and 11 million families in the Syrian Arab Republic (204, 205).

A Generic National Action Plan covering risk assessment, a mechanism for action, determination of roles and responsibilities of each sector, establishment of a coordination mechanism, etc. should be developed. Each province/region should prepare its own plan of action considering local conditions such as epidemiology of EIDs/REIDs, vector and reservoir species and their distribution, response capacity and additional needs, etc.

Even though there have been common risks and drivers for EIDs/REIDs in wars and natural disasters, they have idiosyncratic risks. For example, security is a great problem in wars while the continuity of a natural disaster itself, such as in an earthquake poses, additional risks in implementing control measures.

In order to increase response capacity, planning and investments including awareness raising additional to finance are needed for disaster preparedness and early warning. The establishment or adaptation of the current EIDs/REIDs Surveillance and Early Warning System covering the probable effects of wars and natural disasters on EIDs/REIDs is a prerequisite for recognizing disease clusters/outbreaks, and monitoring and evaluating control interventions. Being an important part of a strong surveillance system, enhanced laboratory services should be planned to include mobile even field laboratories which are capable of sustaining their work under inappropriate conditions in order to provide rapid diagnosis of diseases. Maintenance of surveillance and control activities, waste management services, and food security are of great importance during and after wars and natural disasters in order to reduce their impact on EIDs/REIDs. Given the risk of EIDs/REIDs is much higher in overcrowding and unsanitary conditions, priority should be given to the displaced population and refugees living in temporary shelters and regular screenings can be performed especially for vector-borne diseases such as malaria and leishmaniasis.

Free roaming animals, whether domestic/wild or stray/owned, should definitely be considered, and special control interventions should be implemented as most of the EIDs/REIDs have zoonotic origin. Planning of vector and rodent control is of great importance too. The risk of occurrence of natural disasters and, EIDs/REIDs following natural disasters and during conflict conditions are increasing due to climate change which contributes to creating environmental changes especially favorable for vector-borne diseases.

Therefore, the impact of wars and natural disasters on EIDs/REIDs should be taken seriously. Recommended actions in order to prevent and control EIDs/REIDs in wars and natural disasters are summarized in Table 4.

**Table 4:** Recommended actions in order to prevent and control EIDs/REIDs in wars and natural disasters.

Action	Recommendations
Development of a generic national action plan	Risk assessment for different scenarios (conflict, earthquake, flood, etc.) Mechanism for action Determination of roles and responsibilities of each sector Establishment of a coordination mechanism Standard algorithms for EIDs/REIDs prevention and control Mobile teams for active case detection such as malaria, cutaneous leishmaniasis Coordination with Non-Governmental Organizations and international agencies (WHO, ECDC, etc.) Defining supply management system (laboratory equipment and kits, treatment drugs, vaccine, vector and rodent control equipment and products, etc.), etc.
Preparation of provincial/regional action plans	Consideration of local conditions such as epidemiology of EIDs/REIDs Specification of vector and reservoir species and their distribution Assessment of response capacity and additional needs
Establishment or adaptation of the current EIDs/REIDs surveillance and early warning system	Flexible surveillance system (computerized, paper-based, etc.) Determination of data sources Syndromic surveillance Enhanced laboratory services Mobile laboratories Field laboratories Special arrangements to specimen transport
Environmental health measures	Supply of safe drinking water Basic sanitation facilities
Planning of vector and rodent control	Regularly disposal of excreta, wastewater, and solid wastes Vector control in the affected area Rodent control in the affected area especially around the temporary shelters
Planning of free-roaming-animal control	Planning of new settlements far from wildlife Shelters for domestic animals Vaccination, anti-parasite medication, etc. services to animals hosted in shelters
Arising awareness and capacity building	Training of health staff Public awareness and education programs

## Author contributions

ST, AT-O, and EA: conceptualization, writing—review and editing, and visualization. ST and AT-O: methodology. AT-O and EA: validation. ST: resources and writing—original draft preparation. EA: supervision. All authors contributed to the manuscript development and approved the final version to be published.

## Funding

This study was supported by TOBB-ETU.

## Conflict of interest

The authors declare that the research was conducted in the absence of any commercial or financial relationships that could be construed as a potential conflict of interest.

## Publisher’s note

All claims expressed in this article are solely those of the authors and do not necessarily represent those of their affiliated organizations, or those of the publisher, the editors and the reviewers. Any product that may be evaluated in this article, or claim that may be made by its manufacturer, is not guaranteed or endorsed by the publisher.
